# Steady-State Piperacillin Concentrations in the Proximity of an Orthopedic Implant: A Microdialysis Porcine Study

**DOI:** 10.3390/antibiotics12030615

**Published:** 2023-03-20

**Authors:** Johanne Gade Lilleøre, Andrea René Jørgensen, Martin Bruun Knudsen, Pelle Hanberg, Kristina Öbrink-Hansen, Sara Kousgaard Tøstesen, Kjeld Søballe, Maiken Stilling, Mats Bue

**Affiliations:** 1 Department of Clinical Medicine, Aarhus University, 8200 Aarhus, Denmark; 2 Aarhus Denmark Microdialysis Research Group (ADMIRE), Aarhus University Hospital, 8200 Aarhus, Denmark; 3 Department of Infectious Diseases, Internal Medicine, Gødstrup Hospital, 7400 Herning, Denmark; 4 Department of Orthopedic Surgery, Aarhus University Hospital, 8200 Aarhus, Denmark

**Keywords:** microdialysis, piperacillin, tazobactam, steady state, implants, osteomyelitis

## Abstract

Implant-associated osteomyelitis is one of the most feared complications following orthopedic surgery. Although the risk is low, sufficient antibiotic protection of the implant surface is important. The aim of this study was to assess steady-state piperacillin concentrations in the proximity of an orthopedic implant. Time above the minimal inhibitory concentration (*f*T>MIC) was evaluated for MIC of 8 (low target) and 16 μg/mL (high target). Six female pigs received an intravenous bolus infusion of 4 g/0.5 g piperacillin/tazobactam over 30 min every 6 h. Steady state was assumed achieved in the third dosing interval (12–18 h). Microdialysis catheters were placed in a cannulated screw in the proximal tibial cancellous bone, in cancellous bone next to the screw, and in cancellous bone on the contralateral tibia. Dialysates were collected from time 12 to 18 h and plasma samples were collected as reference. For the low piperacillin target (8 µg/mL), comparable mean *f*T>MIC across all the investigated compartments (mean range: 54–74%) was found. For the high target (16 µg/mL), *f*T>MIC was shorter inside the cannulated screw (mean: 16%) than in the cancellous bone next to the screw and plasma (mean range: 49–54%), and similar between the two cancellous bone compartments. To reach more aggressive piperacillin *f*T>MIC targets in relation to the implant, alternative dosing regimens such as continuous infusion may be considered.

## 1. Introduction

Implants have become a cardinal component of modern orthopedic surgery [1]. The assortment of implants is constantly developing and improving to enrichen treatment options and better patient outcome [2,3]. The most feared complication following orthopedic surgery using implants remains implant-associated osteomyelitis (IAO), accounting for approximately 1–5% of all health care-associated infections in the USA [4,5]. The risk of IAO following orthopedic surgery depends on the type of surgical intervention and geographic location. Although the rate of infection following primary arthroplasty surgery (≥2%), and fracture fixation devices (5%) remains low [1,6], some clinical situations, such as open fractures, have increasing infection rates of up to 30% [7]. The complications following IAO are severe, and include pain, reduced functional outcome, risk of amputations, multiple hospital admissions, complex and repeated surgery, and thus have high costs for the patient as well as the health care system [8,9,10,11]. For infected joint prostheses, the annual economic disbursement of IAO in the USA is estimated to be USD 1.8 billion [12]. Most IAO are caused by staphylococci, however, an increased rate of infection by Gram-negative bacteria has been shown in the 21st century [13,14].

Implantation of a foreign material increases bacterial virulence, as fewer bacteria colony forming units are required to induce a manifest infection compared to tissues free of implants [15,16]. Therefore, all measures must be taken to prevent IAO development, including application of relevant prophylactic antibiotic dosing regimens [15,17,18]. To protect the implant surface and ensure tissue integration, it is crucial to achieve adequate antibiotic concentrations proximate to the implant for a sufficient amount of time.

Piperacillin is a broad-spectrum β-lactam antibiotic, often administered in combination with the β-lactamase inhibitor, tazobactam. The antibacterial activity of piperacillin is time-dependent; hence, the effect is best correlated with the time the concentration of the free unbound drug is maintained above the minimum inhibitory concentration (*f*T>MIC) at the target site [19,20]. Piperacillin/tazobactam (pip/tazo) is not the first choice for orthopedic surgical prophylaxis and treatment of IAO. It is, however, considered effective and often used towards Gram-negative infections [21,22,23]. Gram-negative orthopedic infections have been increasing during the past decades [13,14], wherefore it is of utmost interest to establish optimal dosing regimens based on piperacillin target site *f*T>MIC. As the introduction of implants in bone tissue causes microstructural bone damage affecting the local extracellular environment, it is important to assess the effect of these mechanisms on proximate implant piperacillin concentrations [17,21,23]. This evaluation has previously been methodologically challenging. However, the pharmacokinetic sampling tool microdialysis has proven favorable, given its abilities of providing dynamic sampling of unbound piperacillin concentrations simultaneously from selected target sites [20,24].

In this study, we used microdialysis to assess piperacillin steady-state *f*T>MIC in the proximity of an orthopedic implant following intravenous bolus infusion in pigs. A cannulated screw was applied, giving the opportunity to evaluate concentrations from both the inner and outer layer of the implanted screw.

## 2. Results

All six pigs completed the study. With the exception of one cancellous bone catheter next to the screw, data were obtained from all the catheters. The mean relative recovery (SD) was 27.5% (7.5) for the cannulated screw, 38.5% (15.9) for the cancellous bone next to the screw, and 59.8% (17.7) for the contralateral cancellous bone.

### 2.1. fT>MIC

Mean *f*T>MIC values (minutes and percentages) for all compartments are shown in Table 1. For the low MIC target of 8 μg/mL, no differences were found between all the investigated compartments. For the high MIC target of 16 μg/mL, the *f*T>MIC was shorter for the cannulated screw compared to the cancellous bone next to the screw and plasma.

### 2.2. Pharmacokinetic Parameters

**Figure 1:**
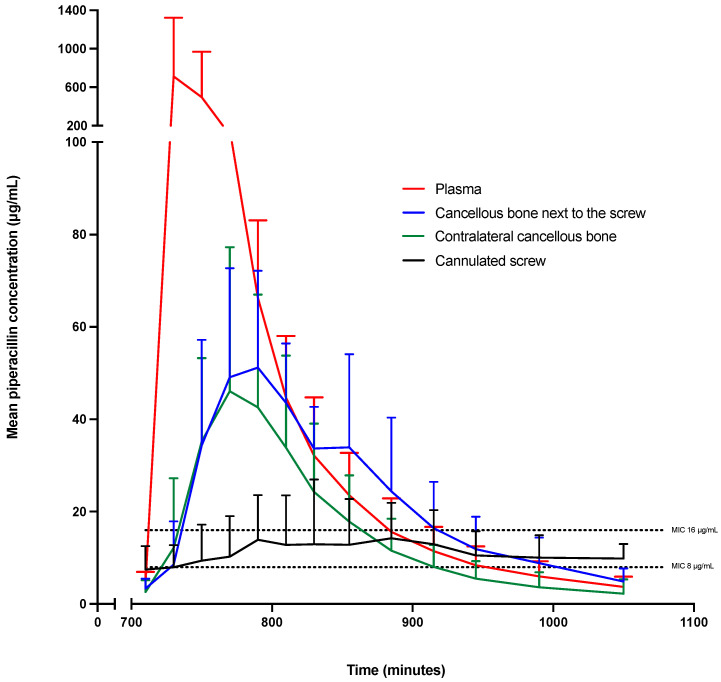
Mean concentration–time profiles of piperacillin in the cannulated screw, the cancellous bone next to the screw, in the contralateral tibia and plasma for the third dosing interval (steady-state concentration). The error bars represent 95% confidence intervals. The left Y-axis shows a two-segmented axis in order to contain all graphs. The X-axis also shows a two-segmented axis to illustrate the true time for sampling.

The calculated pharmacokinetic parameters are presented in Table 2, and the corresponding concentration–time profiles are depicted in Figure 1. Median plasma AUC_12–18 h_ and C_max_ were significantly higher, and plasma T_max_, and T_1/2_ were significantly shorter than in the remaining compartments, except for T_1/2_ for contralateral cancellous bone. Additionally, the cannulated screw differed from the remaining compartments (lower C_max_ and longer T_1/2_). T_1/2_ for the contralateral cancellous bone was significantly lower compared to cancellous bone next to the screw.

## 3. Discussion

We investigated piperacillin steady-state concentrations following bolus administration in the proximity of an orthopedic implant in the form of a cannulated screw, allowing the evaluation of concentrations from both the inner and outer layer of the screw. For the low piperacillin target (8 µg/mL), we found comparable mean *f*T>MIC across all the investigated compartments (mean range: 54–74%), while mean *f*T>MIC for the high target (16 µg/mL) was shorter inside the cannulated screw (mean: 16%) than in the cancellous bone next to the screw and plasma (mean range: 49–54%), and comparable between the two cancellous bone compartments.

The cannulated screw demonstrated significantly lower median C_max_ than all the remaining compartments and the lowest AUC_12–18 h_, but it presented comparable mean *f*T>MIC for the low target (8 µg/mL) and only shorter mean *f*T>MIC for the high target (16 µg/mL) compared to cancellous bone next to the screw and plasma. Pharmacokinetically, this is mainly explained by a fast penetration (short T_max_), mean C_max_ above 8 μg/mL, and a longer elimination (T_1/2_) compared to plasma and the cancellous bone compartments. A prolonged elimination is favorable for time-dependent antibiotics such as piperacillin as it prolongs target exposure. However, in a mechanical deadspace, as in the hollow center of a cannulated screw, a complex situation may arise since the immune system cannot be expected to assist the antibiotic effect as in normally blood perfused tissue. A deadspace is presumably a poorly vascularized void with low oxygen tension and pH, which may favour bacterial proliferation and biofilm formation [25]. A recent microdialysis study has demonstrated the same tendencies of longer elimination of cefuroxime from a physiological hematoma deadspace, indicating longer diffusion distance and lower antibiotic turn-over in a deadspace [26]. Although being a mechanical deadspace, the present experimental design using a cannulated screw reflects that of several clinically relevant orthopedic settings. It is important to bear in mind that assessment of the antibiotic protection of the inner layer of the cannulated screw is merely based on a theoretical discussion and targets and needs further investigations in larger-scale studies.

For evaluation of antibiotic concentration and protection of the outer layer of an implant, the current setup is expected to mimic the true impact of screw insertion on microstructural bone damage and alterations on local perfusion, extracellular pressure, osmotic stress and inflammation. Interestingly, the comparable mean *f*T>MIC piperacillin between the cancellous bone next to the screw and the contralateral tibia, did unexpectedly indicate that *f*T>MIC piperacillin is not affected by the screw-induced stress in the cancellous bone. In contrast to this, a recent study with a comparable setup, investigating co-administered vancomycin and meropenem concentrations in relation to a cannulated screw, suggested that the presence of the screw lowered the concentrations next to the screw in comparison to the contralateral cancellous bone, especially for vancomycin [25]. The investigated drugs differ in molecular sizes, chemical structure and protein binding, but the key difference between the two study setups is the timing of the sampling interval: first dosing interval versus third dosing interval (steady state) in the present study. Studies concerning traumatic bone tissues have described an acute decrease in bone blood flow within the first hours; however, during the first day, blood flow within the traumatic bone rapidly increases three to six times due to angiogenic protective mechanisms [27,28]. As such, the local blood flow surrounding an implant may be a dynamic process regulated within hours to days. The achievement of steady state may therefore explain the different findings between the vancomycin/meropenem study and the present study. This underlines the need of future studies investigating the many aspects and mechanisms in relation to an implant surface and antibiotic protection.

IAO involves complex interactions between the bacteria, the implant (biofilm formation), the antibiotic, and the host immune system [5,19]. When introducing foreign objects, increase in bacterial virulence and the ability of biofilm formation on the implant surface [15,18,29] can develop as early as within the first 24 h after surgery [30]. These factors, along with the vulnerability due to local immune depression in the interstitial environment surrounding the implant [31], may indicate that the role of adequate antibiotic regimens and maintenance of sufficient antibiotic concentrations is particularly important to protect the implant from bacteria colonization.

It may require higher antibiotic concentrations than the employed planktonic targets to gain full antibiotic protection of the implant in orthopaedic surgery. The optimal piperacillin *f*T>MIC target to protect an implant remains unknown. In clinical settings not involving implant surgery, different approaches have been suggested, ranging from 40 to 70% *f*T>MIC in non-critically ill patients, to 100% *f*T>MIC or even 100% *f*T>5xMIC in critically ill patients [32,33]. In the present study, the cannulated screw (inner layer protection) demonstrated a mean *f*T>MIC value of 55% and 16% for the low and high MIC target, respectively, while the cancellous bone next to the screw (outer layer protection) presented with 74% and 54%, respectively. To reach more aggressive targets in relation to the implant, a different dosing strategy may be needed. In the present study, the cannulated screw showed a prolonged T_1/2_ which is favorable as it prolongs target exposure, and thereby mimics continuous infusion to some extent. For piperacillin, application of continuous infusion has been thoroughly investigated, both in terms of clinical outcome and target tissue *f*T>MIC, demonstrating superior effect of continuous infusion [20,34]. Therefore, in high-risk patients and surgeries, and when piperacillin is indicated, continuous infusion may be considered.

The main limitation in the present study was the low number of pigs (n = 6), which may affect study power. However, the frequent microdialysis sampling fully utilizes the potential of the setup and increases the reliability of the reported data. For clinical relevance, piperacillin was administered in combination with tazobactam; however, tazobactam concentrations were not obtained. Previous studies have shown a comparable pharmacokinetic profile of piperacillin when administered alone and together with tazobactam [35,36]. Therefore, no effect of tazobactam on the present piperacillin pharmacokinetic results were assumed [37]. Additionally, precautions should be taken regarding the translational potential of the results due to clinical differences between pigs and humans. Even though pigs and humans resemble each other, to a great extent, in terms of anatomy and physiology, the surgery and following sampling was performed in juvenile (aged 5 months) cancellous bone tissue. Moreover, the present study was performed in healthy, non-inflamed, and non-infected tissue, unlike many other clinical settings.

## 4. Materials and Methods

This study was performed at the Institute of Clinical Medicine, Aarhus University Hospital, Aarhus, Denmark. Chemical analyses were performed at the Department of Clinical Biochemistry, Aarhus University Hospital, Aarhus, Denmark. The study was conducted in accordance with ARRIVE and approved by the Danish Animal Experiments Inspectorate (License No. 2017/15–0201-01184) and carried out in accordance with existing laws and guidelines. To meet the 3 Rs by reducing the number of animal used, the same pigs have provided data to another study with a different purpose [20].

### 4.1. Microdialysis

Microdialysis is a catheter-based method with a precision pump connected to the inlet of the catheter which has a semipermeable membrane at the tip. At the outlet of the catheter, there is a collecting vial. Sampling is based on passive diffusion of unbound molecules across the membrane along the concentration gradient [38]. As the precision pump continuously perfuses the catheter with a low flow rate, complete equilibrium between the inside of the membrane and the surrounding medium cannot occur. Therefore, the dialysate concentration only represents a fraction of the absolute tissue concentration. This fraction is referred to as the relative recovery. To estimate absolute concentrations, determining the relative recovery is important, and can be calculated using different calibration methods [39]. In this study, the internal standard calibration method was applied, using benzylpenicillin as the internal calibrator for piperacillin. The suitability of benzylpenicillin has previously been thoroughly investigated both in vitro and in vivo [24]. All microdialysis equipment was acquired from M Dialysis AB (Stockholm, Sweden) and consisted of 63 microdialysis catheters with a membrane length of 30 mm (20 kDa cut-off), and 107 microdialysis pumps set at a flow rate of 2 µL/min. 

### 4.2. Anesthehsia and Surgical Procedures 

**Figure 2:**
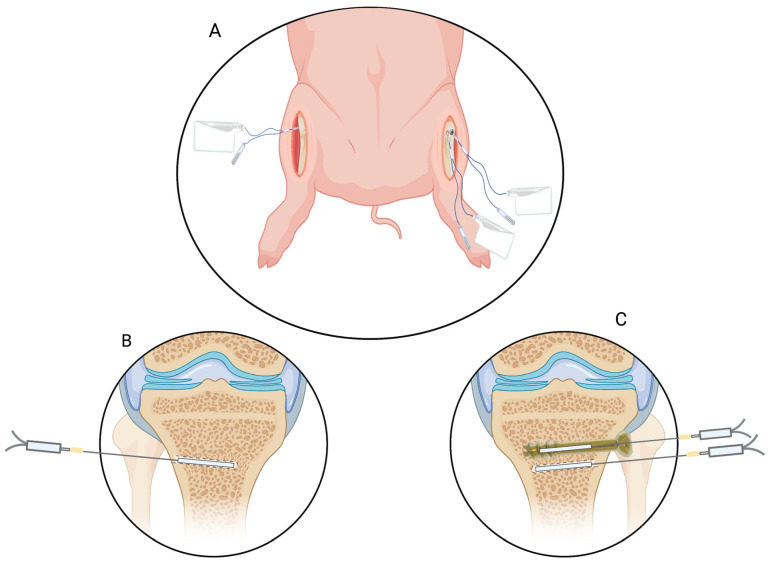
(**A**), illustrative drawing of the placement of the microdialysis catheters in the proximal left and right tibial bone. (**B**), microdialysis catheter placed in a drill hole in the cancellous bone in the contralateral right tibia. (**C**), left tibia: a microdialysis catheter placed in the cannulated screw, and a microdialysis catheter plasced in a drill hole in the cancellous bone approximately 2–4 mm next to the cannulated screw in parallel direction in the axial plane. The illustrated frontal plane in C is for exemplification. Figure 2 was made using Biorender with permission for publication from Biorender [40].

Six female pigs (Danish Landrace Breed, weight 86–90 kg) were included. The pigs were anesthetized by a combination of propofol (B. Braun, Melsungen, Germany) (550 mg/h) and fentanyl (Fresenius Kabi, Bad Homburg, German) (0.6 mg/h). Temperature (range 36.3–38.3 °C) was monitored by a rectal thermometer and regulated with blankets or icepacks. pH (range 7.38–7.53) was monitored through arterial puncture and regulated through ventilation. With the pig in a supine position, the proximal part of the left tibia was assessed through an anteromedial incision. By fluoroscopic guidance, a Kirshner wire (K-wire) (Ø 2 mm, depth 35 mm) was drilled into the cancellous bone distal to the epiphysial plate. The K-wire placed in the left bone was used as guidance to drill a 35 mm hole performed by a cannulated drill. Afterwards, the cannulated drill and K-wire was removed in order to insert a 35 mm cannulated screw (Ø 6.5). Approximately 2–4 mm next to the cannulated screw, a cancellous drill hole was performed in a parallel direction in the axial plane (Ø 2 mm, depth 35 mm). A similar drill dole was performed on cancellous bone in the contralateral leg (Figure 2). To avoid displacement of the catheters, fixation to the skin was performed by a single suture. Correct location of all catheters was ensured by intraoperative fluoroscopy. All animals were euthanized at the end of the sampling period by an intravenous overdose of pentobarbital (Alfasan, Woerden, The Netherlands). 

### 4.3. Drug Administration and Sampling Procedure

**Figure 3:**
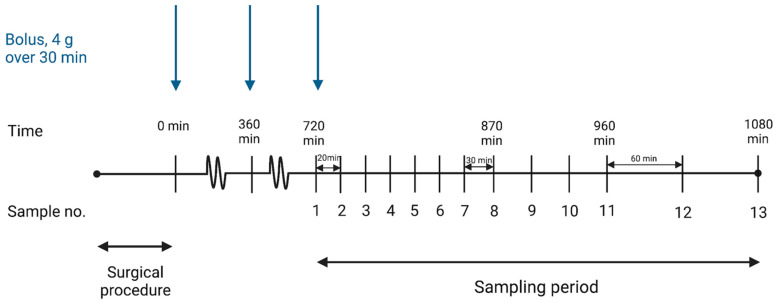
Timeline illustrating the sampling intervals and administration of piperacillin. Figure 3 was made using Biorender with permission for publication from Biorender [40].

Piperacillin was administered as a 4 g bolus infusion in combination with 0.5 g tazobactam (Fresenius Kabi) over 30 min every 6 h. Steady state was assumed achieved in the third dosing interval (12–18 h). A previous study observed a plasma half-life of 74 min (95% CI: 58–90) of piperacillin, suggesting steady state to occur within 5 to 7.5 h [24]. After placement of the microdialysis catheters, they were perfused with 0.9% NaCl containing 5 µg/mL benzylpenicillin. Dialysates were sampled from time 720 to 840 min at 20 min intervals, from time 840 to 960 min at 30 min intervals and from time 960 to 1080 min at 60 min intervals (Figure 3). A pre-dose dialysate sample (720 min) was collected during the 20 min preceding the third dose. In total, 13 samples were collected from each compartment. Venous blood samples were collected from a central venous catheter in the middle of each sampling interval. After collection, dialysates were immediately stored at −80 °C until analysis. Blood samples were stored at 5 °C for a maximum of 4 h before centrifugation at 3000× *g* for 10 min. Afterwards, plasma samples were stored at −80 °C until analysis. 

### 4.4. Quantification of Piperacillin and Benzylpenicillin

Unbound piperacillin concentrations in the microdialysates and plasma samples, and benzylpenicillin concentrations in the microdialysates, were simultaneously quantified using ultra-high performance liquid chromatography with UV detection. For piperacillin, the total imprecisions (CV) were 4% at 8 μg/mL and 2% at 100 μg/mL in 0.9% NaCl solution, and 6% at 5 μg/mL and 9% at 80 μg/mL in plasma. For benzylpenicillin, CV were 5% at 2 μg/mL and 3% at 8 μg/mL in a 0.9% NaCl solution. The lower limit of quantification (LLOQ) was estimated at 0.1 μg/mL (CV% = 18%) for piperacillin and at 0.1 μg/mL (CV% = 11%) for benzylpenicillin. The calibration curve was only accepted if the correlation coefficient was >0.98. A more detailed description of the quantification method has previously been described [20,24].

### 4.5. MIC Targets, Pharmacokinetic Analysis, and Statistics

Microsoft Excel was used to estimate the *f*T>MIC using linear interpolation for each animal and compartment. Piperacillin clinical breakpoint for *Pseudomonas aeruginosa* (16 μg/mL = high target) and Enterobacterales (8 μg/mL = low target), published by the European Committee on Antimicrobial Susceptibility Testing (EUCAST), were used to evaluate *f*T>MIC [41].

Pharmacokinetic parameters were determined separately for each pig and compartment by non-compartmental analysis using STATA (v. 17.0 StataCorp, College Station, TX, USA). Due to the small sample size, normal distribution was not achieved. Therefore, conversion into logarithmic transformation was necessary in order to reduce skewness of the original data. As a result, pharmacokinetic parameters are computed as medians. The following pharmacokinetic parameters were determined: Area under the concentration–time curves (AUC_12–18 h_), peak drug concentration (C_max_), time to C_max_ (T_max_), tissue penetration ratio (AUC_tissue_/AUC_plasma_), and half-life (T_1/2_). AUC_12–18 h_ was calculated using the linear up-log down trapezoidal rule.

C_max_ was calculated as the maximum of all measured concentrations, and T_max_ as the time to reach C_max_. The tissue penetration ratio was estimated as the ratio between AUC_tissue_ and AUC_plasma_. T_1/2_ was calculated as ln (2)/λ_eq_, where λ_eq_ is the terminal elimination rate constant estimated by linear regression of the log concentration on time. All variables were analyzed using a mixed model considering the variances between pigs. The model assumptions were tested by visual diagnosis of residuals, fitted values, and estimates of random effects. A correction for degrees of freedom due to small sample size was performed using the Kenward–Roger approximation method. Overall comparisons between the compartments were assessed using *F*-test followed by pairwise comparison using *t*-test. A *p*-value < 0.05 was considered statistically significant. No correction for multiple comparisons was applied.

## 5. Conclusions

In summary, steady-state concentrations in relation to an orthopedic implant were evaluated for piperacillin therapy regimens. Comparable mean *f*T>MIC was found across all the investigated compartments for the low piperacillin target (8 µg/mL), indicating equal theoretical protection of the inner and outer layer of the screw for this target. For the high target (16 µg/mL), the inner layer of the screw displayed shorter mean *f*T>MIC than in the cancellous bone next to the screw and plasma, and mean *f*T>MIC was comparable between the two cancellous bone compartments. To reach more aggressive piperacillin *f*T>MIC targets in relation to the implant, continuous infusion, or alternative dosing regimens may be considered.

## Figures and Tables

**Table 1:** Mean time above the low and high target MIC for piperacillin presented in percentages and minutes in the third dosing interval (steady-state concentration).

Parameter	Minutes (95% CI)	Percentages (95% CI)
Low target: *f*T>MIC 8 µg/mL		
Cannulated screw	180 (97–263)	55 (29–80)
Cancellous bone next to the screw	243 (151–335)	74 (46–100) *
Contralateral cancellous bone	178 (95–261)	54 (29–79)
Plasma	238 (155–321)	72 (47–97)
High target: *f*T>MIC 16 µg/mL		
Cannulated screw	53 (3–103) ^a^	16 (1–31)
Cancellous bone next to the screw	177 (121–233)	54 (37–71)
Contralateral cancellous bone	112 (62–163)	34 (19–49)
Plasma	162 (111–212) ^a^	49 (34–64)

*f*T>MIC: Time above minimal inhibitory concentration values. ^a^ Comparison of the cannulated screw and the cancellous bone next to the screw and plasma (*p*-value < 0.006). * Values above 100% in 95% CI is set to 100% for clinical applicability.

**Table 2:** Piperacillin pharmacokinetic parameters presented as medians (95% confidence intervals) in the third dosing interval (steady-state concentration).

Parameter	Cannulated Screw	Cancellous Bone Next to the Screw	Contralateral Cancellous Bone	Plasma ^a^
AUC_12–18_ h, min µg/mL	4170 (1315–13,227)	8821 (5511–14,144)	7998 (3896–16,418)	26,167 (14,867–46,055)
C_max_, µg/mL	11 (4–31) ^b^	49 (32–75)	45 (27–77)	571 (215–1522)
T_max_, min ^c^	121 (47–203)	54 (31–78)	53 (38–69)	20 (9–32)
T_1/2_, min	807 (375–1740)	324 (170–618)	131 (79–217) ^d^	65 (39–108)
*f*AUC_tissue_/*f*AUC_plasma_	0.16 (0.07–0.38)	0.35 (0.16–0.79)	0.31 (0.12–0.76)	

^a^ *p*-value < 0.05 for comparison of plasma to all the other bone compartments except for T_1/2_ for the contralateral cancellous bone. ^b^ Comparison of the cannulated screw with both the contralateral cancellous bone; *p*-value = 0.008, and the cancellous bone next to the screw; *p*-value = 0.022. ^c^ T_max_ has been calculated from time 720 min (beginning of the third dosing interval). ^d^ Comparison of the contralateral cancellous bone with both the cannulated screw, *p*-value = 0.001, and the cancellous bone next to the screw, *p*-value = 0.037.

## Data Availability

The data are available on reasonable request from the authors.
